# BJ-1108, a 6-Amino-2,4,5-Trimethylpyridin-3-ol Analog, Inhibits Serotonin-Induced Angiogenesis and Tumor Growth through PI3K/NOX Pathway

**DOI:** 10.1371/journal.pone.0148133

**Published:** 2016-01-29

**Authors:** Suhrid Banskota, Jaya Gautam, Sushil C. Regmi, Pallavi Gurung, Myo-Hyeon Park, Seung Joo Kim, Tae-gyu Nam, Byeong-Seon Jeong, Jung-Ae Kim

**Affiliations:** 1 College of Pharmacy, Yeungnam University, Gyeongsan, 38541, Republic of Korea; 2 Department of Pharmacy, Hanyang University, Ansan, 15588, Republic of Korea; University of Kentucky, UNITED STATES

## Abstract

5-Hydroxytryptamine (5-HT) induces proliferation of cancer cells and vascular cells. In addition to 5-HT production by several cancer cells including gastrointestinal and breast cancer, a significant level of 5-HT is released from activated platelets in the thrombotic environment of tumors, suggesting that inhibition of 5-HT signaling may constitute a new target for antiangiogenic anticancer drug discovery. In the current study we clearly demonstrate that 5-HT-induced angiogenesis was mediated through the 5-HT_1_ receptor-linked Gβγ/Src/PI3K pathway, but not through the MAPK/ERK/p38 pathway. In addition, 5-HT induced production of NADPH oxidase (NOX)-derived reactive oxygen species (ROS). In an effort to develop new molecularly targeted anticancer agents against 5-HT action in tumor growth, we demonstrate that BJ-1108, a derivative of 6-amino-2,4,5-trimethylpyridin-3-ol, significantly inhibited 5-HT-induced angiogenesis. In addition, BJ-1108 induced a significant reduction in the size and weight of excised tumors in breast cancer cell-inoculated CAM assay, showing proportionate suppression of tumor growth along with inhibition of angiogenesis. In human umbilical vein endothelial cells (HUVECs), BJ-1108 significantly suppressed 5-HT-induced ROS generation and phosphorylation of PI3K/Akt but not of Src. Unlike NOX inhibitors, BJ-1108, which showed better antioxidant activity than vitamin C, barely suppressed superoxide anion induced by mevalonate or geranylgeranyl pyrophosphate which directly activates NOX without help from other signaling molecules in HUVECs, implying that the anti-angiogenic action of BJ-1108 was not mediated through direct action on NOX activation, or free radical scavenging activity. In conclusion, BJ-1108 inhibited 5-HT-induced angiogenesis through PI3K/NOX signaling but not through Src, ERK, or p38.

## Introduction

Angiogenesis is the process of generating new blood vessels from pre-existing ones. Normal angiogenesis is a critical process for organ development as well as reproduction and wound healing. However, uncontrolled abnormal angiogenesis is associated with the pathogenesis of various diseases including rheumatoid arthritis, age-related macular degeneration, and diabetic retinopathy [1–3], and is also involved in tumor growth and metastasis. Suppression of such pathological angiogenesis has been a promising approach in prevention and treatment of the diseases.

Cancer and stromal cells in tumor tissues produce a number of angiogenesis inducers including growth factors such as vascular endothelial growth factor (VEGF) and platelet-derived growth factor (PDGF), and cytokines such as interleukin (IL)-8 [4–6]. Similarly, serotonin (5-hydroxytryptamine, 5-HT) is produced from several cancer cells [7, 8], and acts as a mitogenic signal for cancer cells [9–12] and vascular cells [13, 14]. In addition, due to its invasive nature, cancer is associated with thromboembolic complications [15–18], and in such a thrombotic environment, cancer cells activate platelets to release significant levels of 5-HT, leading to direct action in endothelial cells to induce vasoconstriction, platelet thrombus formation [19, 20], and angiogenesis [13].

5-HT induces angiogenic responses of endothelial cells in inflamed vascular tissue such as diabetic blood vessels [13, 21, 22] and in tumor tissues [23, 24], however, the receptor subtype mediating its angiogenic action remains unclear: Mitogenic action of 5-HT in endothelial cells is mediated through 5-HT_2_ [24, 25], whereas 5-HT_1B_-linked angiogenesis occurs in diabetic mice [21]. Likewise, the intracellular signaling molecules linked to angiogenic action of 5-HT are still unclear. Angiogenesis by endothelial 5-HT_1B_ is mediated by the Akt/eNOS pathway in mice [21], whereas 5-HT_2B_ induces ERK activation in its angiogenic response [24]. In addition, 5-HT, similar to VEGF signaling, was reported to induce activation of Src/PI3K/AKT/mTOR/p70S6K as well as ERK and p38 in HUVECs [13].

Reactive oxygen species (ROS) including superoxide anion and hydrogen peroxide (H_2_O_2_) are frequently observed in the processes of tumorigenesis and angiogenesis as well as VEGF expression [26–28]. ROS are also implicated as signaling molecules in 5-HT-induced mitogenesis of smooth muscle cells and cardiac myocytes [29]. Although NADPH oxidase (NOX)-2 is known to be the major ROS source in HUVECs, and involved in VEGF-induced angiogenesis [30], its involvement in 5-HT-mediated angiogenesis has not been studied.

6-Amino-2,4,5-trimethylpyridin-3-ol scaffold was first studied for its antioxidant activity [31, 32]. Based on studies showing that angiogenesis can be inhibited by some antioxidants [33, 34], we recently reported that its analogues have antiangiogenic activity [35]. Structurally diverse mono- and bicyclic analogues were synthesized, many of which showed potent activity. Among them, BJ-1108 is classified as a monocyclic analogue with the simplest phenyl substituent for minimal steric effect and showed comparable antiangiogenic activity to SU-4312, a potent and selective inhibitor of VEGFR, in a VEGF-induced angiogenesis model using Chick Chorioallantoic Membrane (CAM) assay. A previous study reported that 5-HT and VEGF share a common angiogenesis signaling pathway [13], suggesting an inhibitory effect of BJ-1108 on 5-HT-induced angiogenesis. However, even the mechanism of action of BJ-1108 by which it inhibits VEGF-induced angiogenesis was not clearly understood.

In the current study, we not only examined the receptor subtype and detailed signaling pathway mediating 5-HT action in angiogenesis, but also examined the inhibitory effect of BJ-1108, a 6-aminopyridin-3-ol analog, on 5-HT-induced angiogenesis, and its mechanism of action.

## Materials and Methods

### Materials

All chemical reagents were purchased from Sigma-Aldrich (St. Louis, MO, USA) unless specified. The human umbilical vein endothelial cell line (HUVEC), Endothelial cell basal medium-2 (EBM-2), HEPES-buffered saline solution, Trypsin/EDTA, Trypsin Neutralizing solution (TNS), and EGM-2 Single Quots were purchased from Lonza (Walkersville, MD, USA). The human breast cancer cell lines MDA-MB-231 and MCF-7 were purchased from American Type Culture Collection (Manassas, VA, USA). Dulbecco's modified Eagle's medium (DMEM) and RPMI1640 were purchased from Hyclone (Logan UT, USA). Vascular endothelial growth factor (VEGF) and cortisone acetate were purchased from R&D Systems (Minneapolis, MN, USA). Fetal bovine serum (FBS), penicillin, and streptomycin were purchased from Gibco (Grand Island, NY, USA). Antibodies against phospho-src (at Tyr416), src, phospho-p85-PI3K (at Tyr488), p85-PI3K, phospho-AKT (at T308), AKT, phospho-mTOR (at Ser2448), mTOR, phospho-ERK (at T202) ERK, phospho-p38 (at T180), and p38 were purchased from Cell Signaling Technology Inc. (Beverly, MA, USA), Celecoxib, p47phox and β-actin antibodies were purchased from Santa Cruz Biotechnology (Santa Cruz, CA, USA), and phospho-p47phox antibody was obtained from Abcam (Cambridge, MA, USA). Matrigel was purchased from BD Biosciences (Bedford, MA, USA). Gallein, AZM-475271, Cyanopindolol hemifumarate, Cinanserin hydrochloride, and SB-269970 hydrochloride were purchased from Tocris Bioscience (Tocris House, Bristol, BS110QL, UK). Wortmannin was purchased from Biomol (Plymouth Meeting, PA, USA).

### Chick chorioallantoic membrane (CAM) assay

CAM assay was performed as previously described [36, 37]. Briefly, fertilized chicken eggs purchased from Siprigoal Poultry Farm (Gyeongbuk, South Korea) were carefully incubated at 37°C in 55% relative humidity. On the 10^th^ day, the bifurcated vessels were selected and a hypodermic needle was used to make a hole at the wide end of the eggshell concealing the air sac. A second hole was made on the broader part of the egg by applying negative pressure to create a false air sac beneath it in order to separate the membrane with vessel from the shell. A window of approximately 1 cm^2^ was made above the false air sac on the broader side of the shell. A sterile filter disc, pretreated with 3 mg/mL of cortisone acetate and soaked with 5-HT, dissolved in PBS with 1% bovine serum albumin (BSA) was used to stimulate the growth of the blood vessel. After 30 min, 10 μL of BJ-1108 at different concentrations as indicated were topically added directly to the CAMs. The CAM tissue beneath the disk was resected from the embryo after incubation for 72 h and then harvested. Photographs were analyzed using an image software program. The number of branch points sprouting from the original vessels was counted in the circular region of the disk. Results from more than six eggs per treatment group were expressed as the mean ± S.E.M of the number of blood vessel branches formed in each sample set.

In the case of tumor-induced angiogenesis, all procedures were the same except that tumor cells (2×10^6^ cells/CAM) were inoculated onto the CAM with or without compounds instead of the disc containing 5-HT.

Chick embryo experiments were performed in accordance with the institutional guidelines of the Institute of Laboratory Animal Resources (1996) and Yeungnam University for the care and use of laboratory animals (2009). The experiment protocol was reviewed and approved by the Institutional Animal Care and Use Committee at Yeungnam University.

### Cell culture

Human umbilical vein endothelial cells (HUVECs) were maintained in culture flasks coated with 0.2% gelatin in EBM-2 supplemented with EGM-2 Single Quots. Confluent cultured cells from passages 2 to 6 were used. MCF-7 and MDAMB-231 human breast cancer cell lines were maintained in DMEM containing 10% FBS and 1% streptomycin and penicillin.

### Tube formation assay

Tube formation assays were performed as described by Kang et al, 2014 [30]. HUVECs were suspended in EBM-2 containing 1% FBS and plated in 48-well plates pre-coated with Matrigel at a density of 1×10^4^ cells per well with or without 5-HT and indicated compounds. After 18 h, five different fields in each well were randomly selected and photographed under a microscope attached to a CCD camera (TE2000-U; Nikon). The images were analyzed using an image analysis system (Image Inside) for quantitation of tube length.

### Migration assay

A wound healing assay was performed in 6-well plates following the method described by Park et al, 2007 [38]. HUVECs were treated with 25 μg/mL mitomycin C for 30 min before making an injury line with a 1-mm tip width on the cells seeded in wells at 90% confluency. The cells were then rinsed with HBSS and allowed to migrate in the presence of VEGF and 5-HT in complete media and a photograph was captured using a microscope (×200) at 0 h and 5 h.

### ROS Measurement

Intracellular ROS was measured using 2,'7'-dichlorofluorescein diacetate (DCF-DA), a cell-permeable fluorogenic probe, with slight modification as described by Regmi et al., 2014 [39]. HUVECs (0.5×10^5^ cells/cm^2^) seeded in 24-well plates were treated with 5-HT for different time periods. The cells were then washed with PBS and incubated with 10 μM DCF-DA for 30 min at 37°C. After washing with PBS 3 times, the images were captured using a camera (TE2000-U, Nikon, Japan) with a blue filter (B-2E/C, FITC) at magnification of 200X.

Lucigenin chemiluminescence assay was performed as described by Banskota et al. for measurement of superoxide anion [40]. Briefly, HUVECs were seeded in white opaque 96-well plates (0.5×10^5^ cells/well). The next day, the cells were pretreated with drugs for 1 h prior to treatment with or without 5-HT (10 μM) for the indicated time. Chemiluminescence was then measured using lucigenin (400 μM) in a Fluostar Optima microplate reader.

### 1,1-Diphenyl-2-picrylhydrazyl (DPPH) free radical scavenging activity assay

The colorimetric assay was performed to determine the free radical scavenging activity of the compounds. Final dilution of all compounds was prepared in DMSO, and DPPH was dissolved in methanol. To 10 μL of DMSO or drug solution, 190 μL of DPPH solution was added so that the final concentration of DPPH remained 200 μM and the concentration of the compound remained as indicated. The reaction was incubated at 25°C in the dark. Absorbance was then measured using Spectro Star Nano at 517 nm. Free radical scavenging activity of the compound was calculated using the given equation.

Free radical scavenging activity (%) = [(A_C_-A_T_)/A_C_]×100% (A_C_: absorbance of control, A_T_: absorbance of test sample)

### KDR kinase assay

KDR kinase enzyme system (Promega #V2681) and ADP-Glow^™^ kinase assay kit (Promega #V9101) were used in performance of the KDR kinase assay. To a mixture consisting of 1.5 ng/μL of KDR enzyme, 0.2 μg/μL of poly (Glu4, Tyr1) and indicated concentration of drugs, 50 μM of ATP (final concentration) was added to start the reaction. The reaction was carried out for 60 min at room temperature in a total volume of 25 μL. Then, 25 μL of ADP-Glow^™^ reagent was added to the mixture with incubation for 40 min at room temperature. Again after the addition of 50 μL of kinase detection reagent and incubation of the mixture at room temperature for 30 min, the luminescence was recorded using a Fluostar optima microplate reader (BMG Labtech GmbH, Offenburg, Germany).

### Western blotting

HUVECs were pretreated with indicated compounds for 1 h prior to treatment with 5-HT and whole lysates were prepared using RIPA buffer containing proteases and phosphatase inhibitors. BCA protein assay reagent (Pierce, Rockford, IL, USA) was used for quantitation of the protein content. Equal amounts of protein were separated by SDS-PAGE and transferred at 200 mA onto Hybond ECL nitrocellulose membranes (Amersham Life Science, Buckinghamshire, UK) for 1 h. The membranes were blocked in 5% bovine serum albumin (BSA) and 5% skim milk in Tris-buffered saline (TBS)-Tween 20 (TBS-T) for phosphorylated and total protein, respectively, at room temperature for 1 h followed by incubation with specific antibodies in BSA or skim milk-TBS at 4°C overnight. The membrane was then washed three times with TBS-T and incubated with horseradish peroxidase-conjugated secondary antibody in BSA or skim milk-TBS for 1 h at room temperature. Immunoreactive proteins were detected using an ECL kit (Pierce) under a luminescent image analyzer, LAS-4000 mini (Fuji, Japan). The membranes were reprobed with β-actin antibody for loading control. The protein density was analyzed using Multi Gauge Ver 3.2 imaging software in a Fuji Image Station.

### Src kinase assay

The Cyclex c-Src Kinase Assay/Inhibitor screening Kit (# CY-1083) was used to determine c-Src kinase inhibitory activity of chemicals. BJ-1108 was prepared at 10x concentration in distilled water. The kinase reaction was carried out in a tyrosine kinase-substrate-1 pre-coated 96-well plate with 10 μl of recombinant catalytic domain of c-Src, 80 μl of kinase reaction buffer and 10 μl of BJ-1108 (10 μM). c-Src kinase activity was measured calorimetrically at 450 nm using a microplate reader (Versamax, Molecular Devices, Sunnyvale, CA, USA) in accordance with the manufacturer’s instructions.

### PI3K activity assay

PI3Kinase enzyme activity was measured using a PI3Kinase activity/inhibitor assay kit (Millipore) according to the manufacturer’s protocol. BJ-1108 and PIP2 substrate were added to 96-well ELISA plates precoated with glutathione. After incubation at room temperature for 1 h, biotinylated-PIP3/EDTA solution was added to each well for 1 h at room temperature. After washing four times with TBST, streptavidin-HRP solution was added, followed by incubation for 1 h at room temperature. The plates were washed three times with TBS. The TMB substrate was added to each well, followed by incubation for 20 min. Optical density was measured using a microplate reader (450 nm). The relative percentage to biotinylated-PIP3 was calculated as described in the manual.

### Xanthine oxidase (XO) activity assay

Superoxide anion scavenging activity was determined using the xanthine/xanthine oxidase system. All solutions were prepared in phosphate buffer pH 7.4 except xanthine, which was first dissolved in 1 M NaOH and subsequently diluted to the required concentration. To 50 μL of vehicle or sample, 50 μL of xanthine (400 μM) and 50 μL of xanthine oxidase (0.4 U/mL) were added in 96-well plates. To start the reaction, 50 μL of 2.4 mM nitro blue tetrazolium (NBT) was added with incubation for 10 min at room temperature. The color intensity of reduced NBT by superoxide anion was measured at 560 nm using Spectro Star Nano. The superoxide scavenging activity was calculated in terms of % inhibition with the following equation. Then the IC_50_ was evaluated.

Inhibition (%) = [(A_C_-A_T_)/A_C_]×100% (A_C_: absorbance of control, A_T_: absorbance of test sample)

### Statistical analysis

Data are presented as Mean ± S.E.M. of three independent experiments. Statistics were analyzed using Student’s t-test or one-way ANOVA followed by the Student-Newman-Keuls comparison method for calculation of differences between groups (GraphPad Prism 5.0 software, San Diego, CA, USA). P values less than 0.05 were considered statistically significant.

## Results

### 5-HT-induced angiogenesis is mediated through 5-HT_1_ receptor and its downstream Gβγ/Src/PI3K pathway, but not through MAPK/ERK/p38

**Fig 1:**
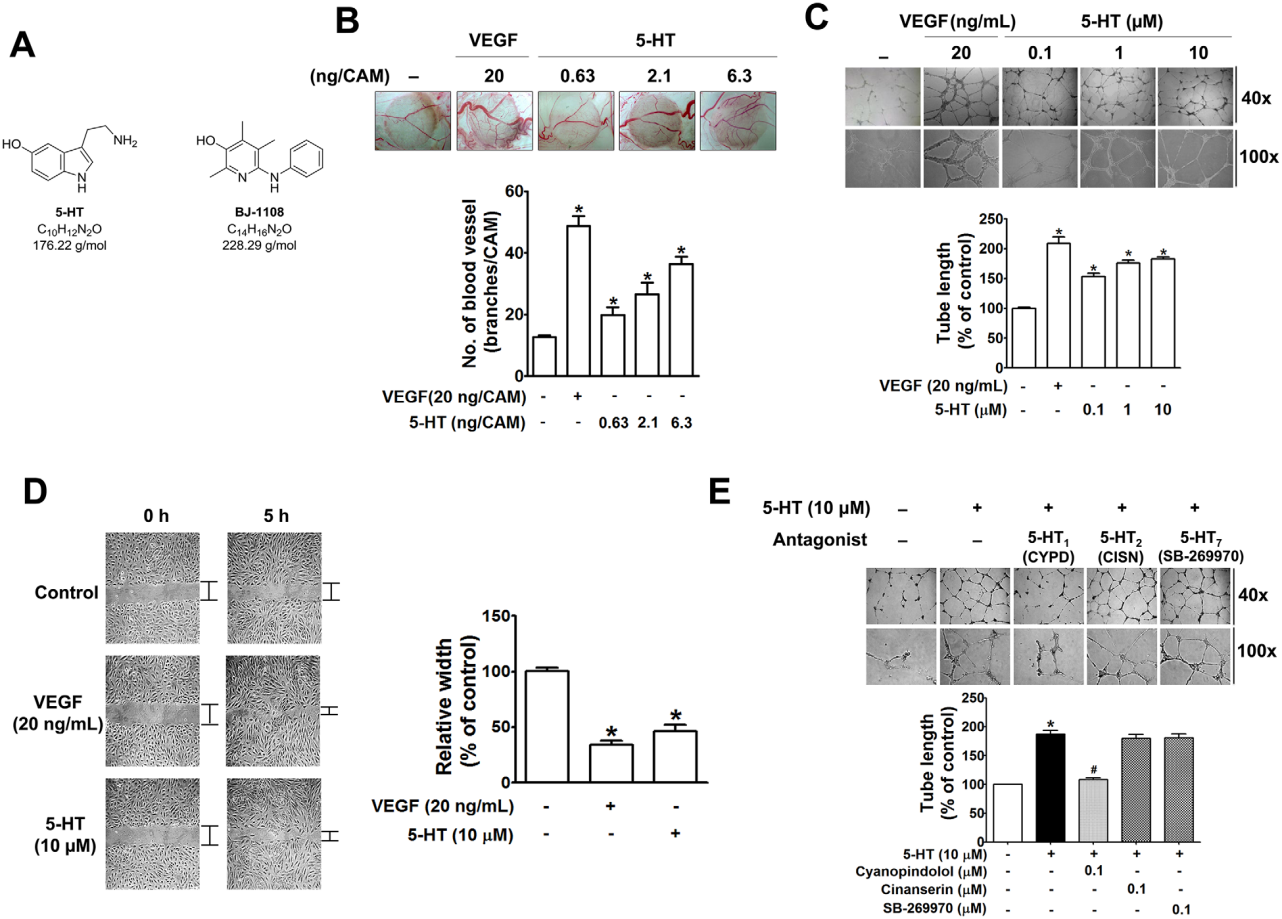
5-HT-induced angiogenesis is mediated through 5-HT1, but not through 5-HT2 and 5-HT7 receptors. (A) Structural formula of 5-HT and BJ-1108. (B) After incubation with 5-HT for 3 days, the newly formed blood vessel branches on the 13-day-old chick CAM were counted. Data represent the mean ± S.E.M. of at least seven chick embryos. *P <0.05, compared with the untreated control group, (C) HUVECs in Matrigel-coated plates were treated with 5-HT for 14 h, and digital images were then taken under the microscope. (D) Migration pattern of HUVECs after treatment with VEGF or 5-HT for 5 h. The bar graph represents the mean ± S.E.M. of at least three independent experiments. *P<0.05, compared with the untreated control group. (E) Tube formation of HUVECs treated with 5-HT for 14 h in the absence or presence of 0.1 μM cyanopindolol (CYPD), Cinanserin (CISN), or SB269970 was quantitated by measuring tube length on the digital images. The bar graph represents the mean ± S.E.M. of at least three independent experiments. *P<0.05, compared with the untreated control group. #*P*<0.05 compared with the 5-HT-stimulated group.

5-HT induced concentration-dependent angiogenesis, as indicated by *in vivo* CAM assay (Fig 1B) and *in vitro* tube formation (Fig 1C). Similar to VEGF, 5-HT stimulated HUVEC migration, which is also one of the angiogenic processes (Fig 1D). To determine a receptor subtype involved in such 5-HT actions mediating angiogenesis, we introduced and compared the effects of receptor antagonists of 5-HT_1_, 5-HT_2_, and 5-HT_7_ on 5-HT-inducced tube formation of HUVECs. Cyanopindolol (5-HT_1A/1B_ antagonist), but not cinanserin (5-HT_2_ antagonist), or SB-269970 (5-HT_7_ antagonist), significantly suppressed 5-HT-induced tube formation (Fig 1E), indicating that 5-HT-induced angiogenesis was 5-HT_1A/1B_ receptor-mediated.

**Fig 2:**
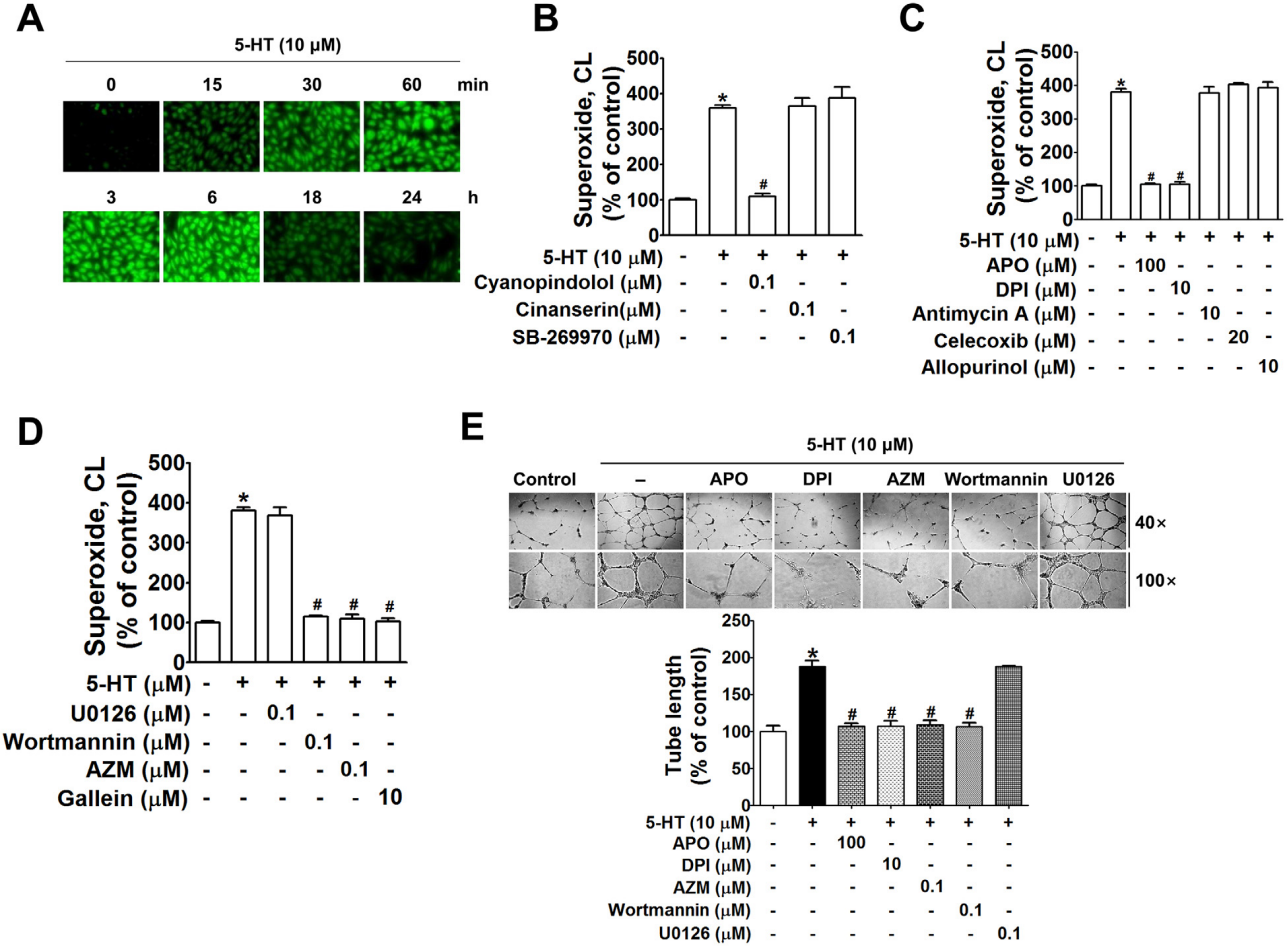
5-HT-induced ROS was suppressible by inhibitors of Gβγ, Src, PI3K, and NOX in HUVECs. (A) Intracellular ROS level detected using DCF-DA in HUVECs treated with 5-HT (10 μM) for the indicated time was determined by fluorescence microscopy. (B-D) Superoxide production was measured by lucigenin chemiluminescence assay. HUVECs treated with 5-HT for 3 h in the absence or presence of 5-HT receptor antagonists (B), various ROS-producing enzyme inhibitors (C), and inhibitors of PI3K, Src and Giβγ (D). (E) Tube formation of HUVECs treated with 5-HT with or without inhibitors against NOX (Apo, DPI), Src (AZM-475271), PI3K (wortmannin), and MAPK (U0126) was quantitated by measuring tube length on the digital images. The bar graph represents the mean ± S.E.M. of at least three independent experiments. *P<0.05, compared with the untreated control group. #*P*<0.05 compared with the 5-HT-stimulated group.

Stimulation of 5-HT_1A/1B_ receptor leads to activation of Gαi and Gβγ, and Gβγ was reported as a signaling molecule in 5-HT-induced angiogenesis (Mukhin et al., 2000). Because Gβγ is known to activate the PI3K signaling pathway, which is, in turn, linked to activation of NOX, in the current study, we attempted to determine whether NOX and NOX-derived ROS are involved in 5-HT-induced angiogenesis signaling. In HUVECs, ROS level was time-dependently increased by 5-HT up to 6 h (Fig 2A). This 5-HT-induced ROS production was suppressed by cyanopindolol, but not by cinanserin and SB-269970 (Fig 2B). In addition, ROS was blocked by apocynin and diphenyleneiodonium chloride (DPI) (NOX inhibitors), but not by antimycin A (mitochondria uncoupling agent), celecoxib (COX-2 inhibitor), or allopurinol (xanthine oxidase inhibitor) (Fig 2C). Inhibitors of Src (AZM-475271), PI3K (wortmannin), and Gβγ (gallein) suppressed 5-HT-induced ROS production, whereas U0126, an inhibitor of MAPK/ERK/p38 kinase, did not suppress 5-HT-induced ROS in HUVECs (Fig 2D). Similar to the effects of signaling inhibitors on 5-HT-induced ROS production, 5-HT-induced tube formation was inhibited by apocynin, DPI, AZM-475271, and wortmannin, but not by U0126 (Fig 2E), indicating that 5-HT-induced angiogenesis was mediated through the Gβγ-activated Src/PI3K/Akt /NOX pathway.

### BJ-1108, a 6-amino-2,4,5-trimethylpyridin-3-ol analog, inhibits 5-HT-induced ROS through 5-HT1-linked signaling pathway

**Fig 3:**
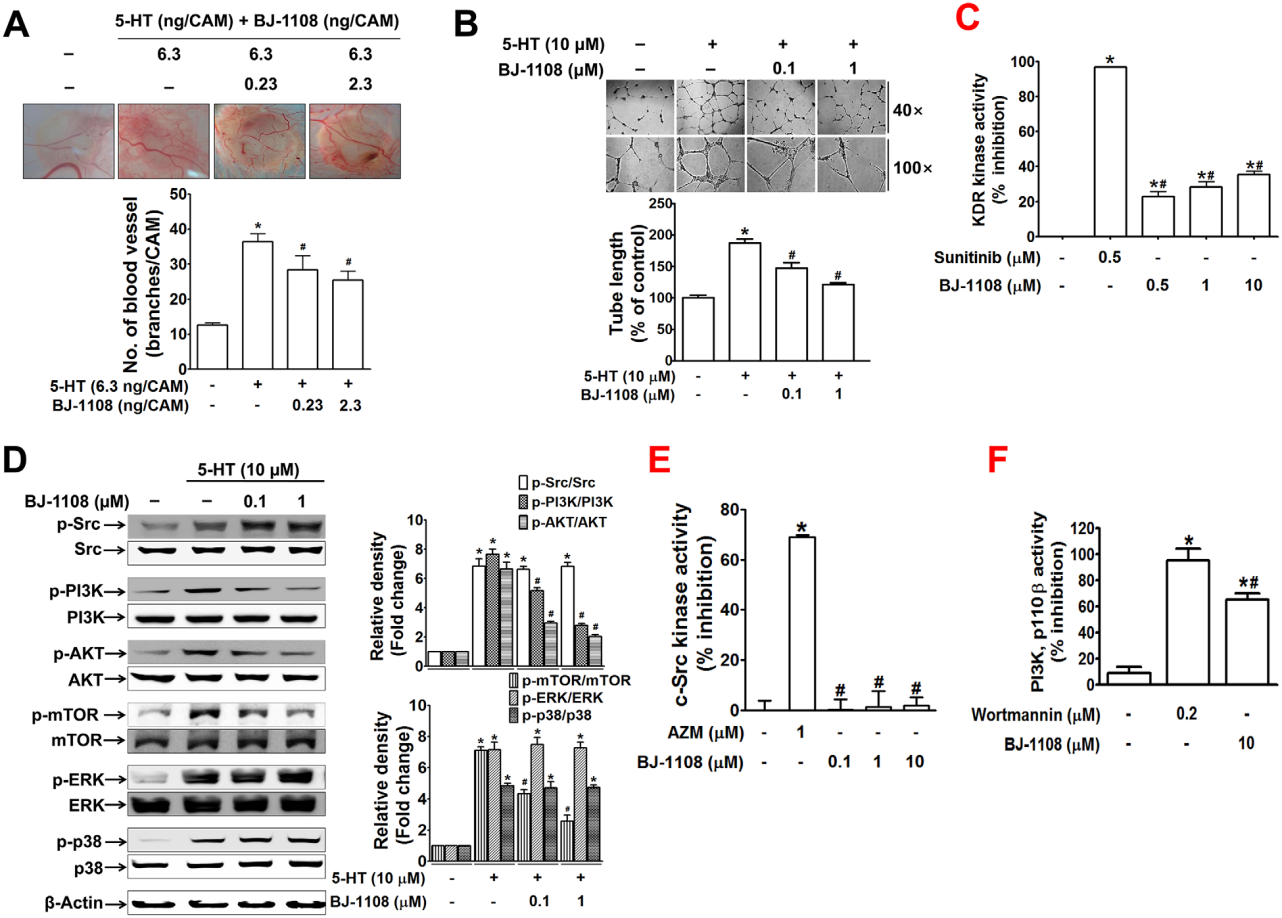
BJ-1108, a novel pyridinol analog, inhibits 5-HT-induced angiogenesis, in a PI3K-dependent manner. (A) The CAM of a 10-day-old chick embryo was treated with 5-HT, and BJ-1108 was applied 30 min later. The bar graph represents the mean ± S.E.M. of at least seven chick embryos. *P <0.05, compared with the PBS-treated control group. #*P*<0.05 compared with the 5-HT-stimulated group. (B) The tube length of HUVECs pretreated with BJ-1108 prior to 5-HT treatment for 14 h was quantitated. (C) KDR (VEGF receptor 2 tyrosine kinase) activity *in vitro* was measured by KDR kinase enzyme system and ADP-Glow^™^ kinase assay kit. Sunitinib malate was used as a positive control. The bar graph represents the means ± S.E.M. of at least three independent experiments. *P<0.05, compared to control and #*P*<0.05, compared to sunitinib. (D) Following pretreatment of HUVECs with BJ-1108 prior to treatment with 5-HT for 5 min, the expression level of phosphorylated form of signaling molecules was analyzed by immunoblotting. Antibodies against phospho-src (at Tyr416), phospho-p85-PI3K (at Tyr488), phospho-AKT (at T308), phospho-mTOR (at Ser2448), phospho-ERK (at T202), and phospho-p38 (at T180), were used for detection of the phospho form of respective proteins. (E) c-Src kinase activity was measured using the Cyclex c-Src Kinase Assay/Inhibitor screening kit. AZM-475271 was used as a positive control. (F) PI3K enzyme activity of the catalytic subunit, p110 was measured using a competitive PI3K activity assay kit, and wortmannin was used as a positive control. The bar graph represents the means ± S.E.M. of at least three independent experiments. *P<0.05, compared to control and #*P*<0.05, compared to wortmannin.

Based on reports that 5-HT and VEGF share a common angiogenesis signaling pathway [13], we next examined the question of whether a compound showing antiangiogenic activity against VEGF could also inhibit 5-HT-induced angiogenesis. Because it possessed a strong inhibitory activity against VEGF-induced angiogenesis [35], BJ-1108 (2,4,5-trimethyl-6-phenylaminopyridin-3-ol) was selected and its inhibitory effect on 5-HT-induced angiogenesis was evaluated. In the CAM assay (Fig 3A) and *in vitro* tube formation of HUVECs (Fig 3B), 5-HT-induced angiogenesis was significantly inhibited by BJ-1108 in a concentration-dependent manner. Although strong inhibition of VEGF-induced angiogenesis by BJ-1108 was reported in the previous study [35], in the current study, it was identified as a much weaker inhibitor of VEGF receptor tyrosine kinase than sunitinib, a pan-receptor tyrosine kinase inhibitor (Fig 3C). These results indicate that BJ-1108 acted on a down-stream signaling molecule to a receptor tyrosine kinase that is common to VEGF and 5-HT. In Western blot analyses, BJ-1108 inhibited 5-HT-induced phosphorylation of PI3K and its down-stream molecules, Akt, and mTOR (Fig 3E). Interestingly, treatment of HUVECs with BJ-1108 did not block 5-HT-induced phosphorylation of Src. Also, 5-HT-induced ERK and p38 phosphorylation was not inhibited by BJ-1108 (Fig 3E). We further examined direct inhibitory activity of BJ-1108 against Src and PI3K kinase activation using an individual assay kit. Compared to positive controls, AZM-475271 and wortmannin, for Src and PI3K, respectively, BJ-1108 had no inhibitory effect on c-Src kinase activity (Fig 3F) but suppressed the activity of p110β, the catalytic subunit of class I PI3K (Fig 3G).

**Fig 4:**
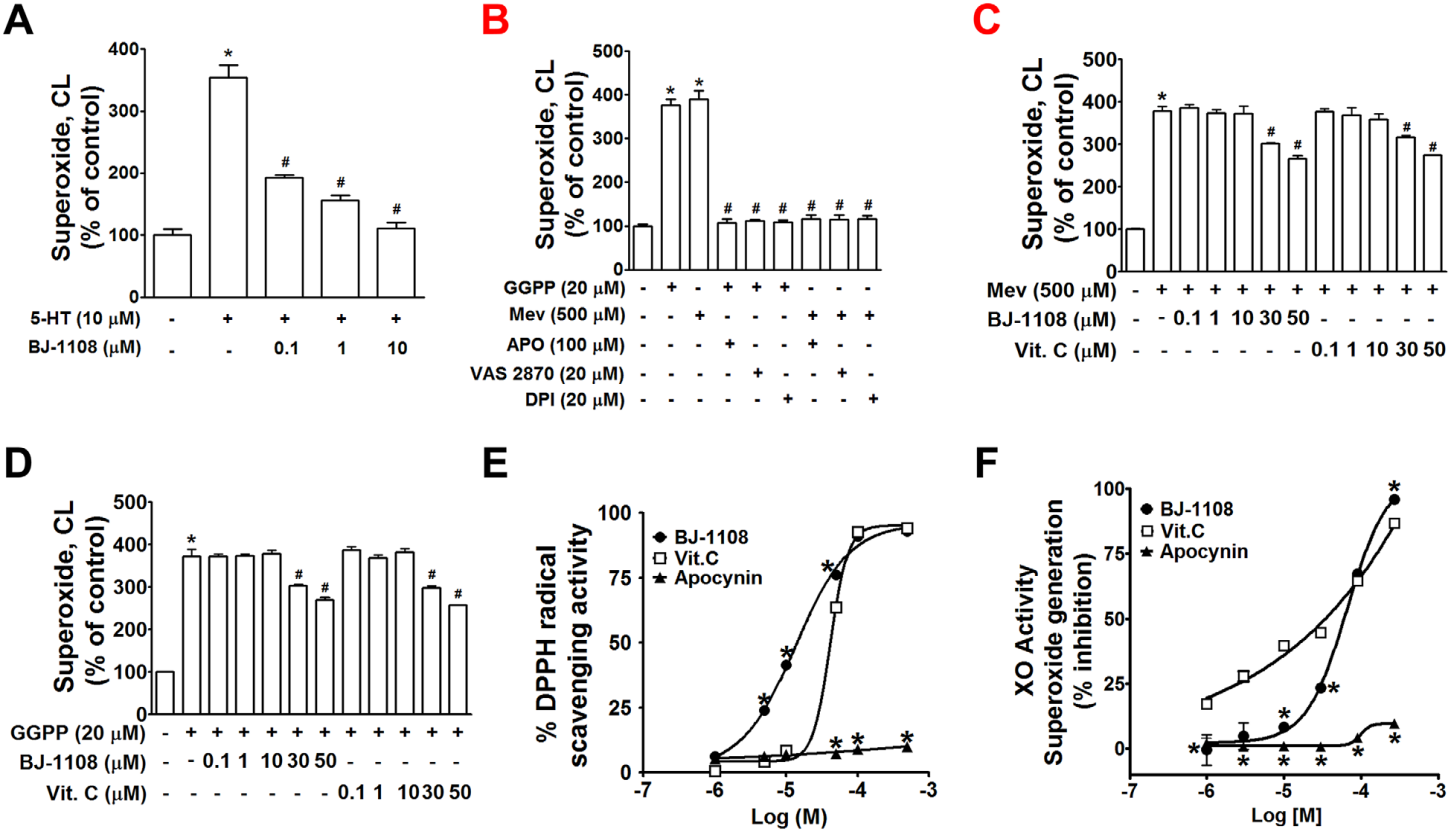
Inhibitory effect of BJ-1108 on 5-HT-induced superoxide was not due to antioxidant activity or direct inhibitory action on NOX activation. (A) Superoxide production was measured by lucigenin chemiluminescence assay in HUVECs pretreated with BJ-1108 for 1 h prior to 5-HT for 3 h. (B-D) HUVECs were treated with mevalonate (MEV, 500 μM), or geranylgeranyl pyrophosphate (GGPP, 20 μM) for 3 h. Before treatment with MEV or GGPP, HUVECs were pretreated with NOX inhibitors (apocynin, VAS2870, DPI) (B), and BJ-1108 or Vitamin C (C, D) for 1 h. The bar graph represents the mean ± S.E.M. of at least three independent experiments. *P<0.05, compared to control and #*P*<0.05, compared to MEV or GGPP. (E) Comparison of DPPH radical scavenging activity of BJ-1108, Vitamin C, and apocynin. (F) Comparison of XO-derived superoxide scavenging activity of BJ-1108, Vitamin C, and apocynin. Data shown are expressed as the mean ± SEM of three independent experiments. *P<0.05, compared to Vitamin C.

Next, we examined the question of whether BJ-1108 can inhibit 5-HT-induced ROS generation, and whether the inhibitory effect is mediated through the PI3K/NOX pathway or by direct inhibition of NOX activation. 5-HT-induced ROS production was inhibited by BJ-1108 in a concentration-dependent manner with almost complete suppression at 10 μM (Fig 4A) in HUVECs. We then attempted to determine whether the inhibitory effect of BJ-1108 on 5-HT-induced ROS was due to its inhibition of a signaling pathway linked to NOX-derived ROS production, or simple antioxidant activity. First, to examine the possibility of BJ-1108 having direct inhibitory action in NOX activation not linked to PI3K, we used mevalonate (Mev) or geranylgeranyl pyrophosphate (GGPP), which directly activates NOX, enerating ROS through post-translational modification of Rac [41]. This Mev- or GGPP-induced ROS was completely blocked by NOX inhibitors DPI, apocynin, and VAS2870 (Fig 4B), whereas it was not blocked by low concentrations (up to 10 μM) of BJ-1108 but blocked by high concentrations (30–50 μM) of BJ-1108 (Fig 4C and 4D). In fact, BJ-1108 showed greater ability to scavenge DPPH radical than vitamin C (Fig 4E, Table 1). However, BJ-1108 was less effective than vitamin C in scavenging superoxide radical generated by xanthine oxidase (Fig 4F, Table 1). These results imply that the inhibitory effect of BJ-1108 on 5-HT-induced ROS was not associated with its antioxidant activity or direct NOX inhibition. Rather, it was mediated through inhibitory action in the Gβγ/PI3K/Akt pathway.

**Table 1:** Comparison of free radical scavenging activities of BJ-1108, vitamin C, and apocynin.

	IC_50_ (μM)	
	DPPH radical scavenging activity	XO-derived superoxide scavenging activity
BJ-1108	14.5	60.3
Vitamin C	38.0	33.9
Apocynin	> 1000	> 1000

### BJ-1108 inhibits tumor angiogenesis and breast cancer growth

**Fig 5:**
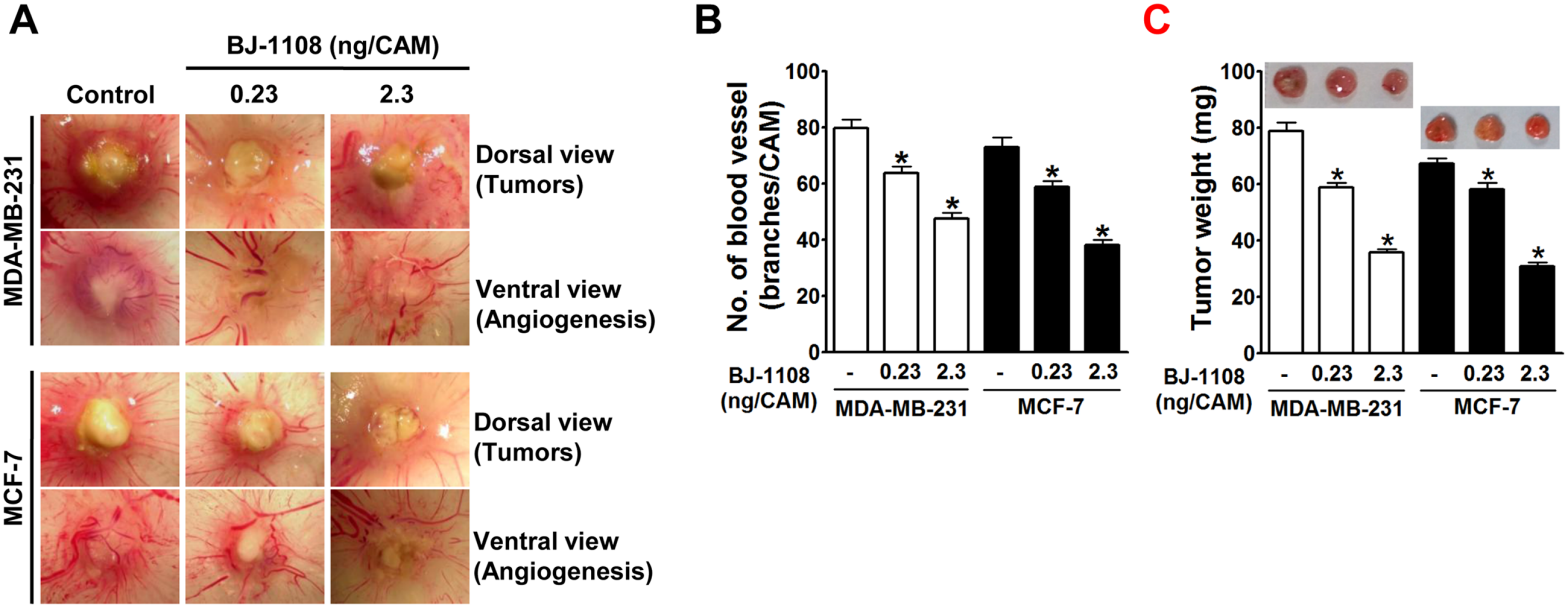
Inhibitory effects of BJ-1108 on human breast cancer cells (MDA-MB-231 and MCF-7)-induced angiogenesis and tumor growth. MDA-MB-231 and MCF-7 human breast cancer cells (2×10^6^ cells/CAM) were inoculated on top of CAM, and BJ-1108 was mixed with cell suspension before inoculation. (A) Digital images of CAM sections were captured, and (B) imported into the image analysis program for quantitation of the number of new vessel branches formed. (C) Tumor mass was isolated and weighed. Data represent the mean ± S.E.M. of at least seven chick embryos. *P<0.05, compared with vehicle-treated control.

Next, potential of BJ-1108 as an antiangiogenic and antitumor agent was examined using cancer cell-inoculated CAM assay. Based on reports that increased biosynthetic capacity of 5-HT was observed in human breast cancer cells [7], we selected MCF-7 and MDA-MB-231 human breast cancer cell lines for demonstration of antitumor activity of BJ-1108. MCF-7 and MDA-MB-231 human breast cancer cells implanted and grown on top of CAM developed a tumor mass (Fig 5A). Treatment with BJ-1108 resulted in significantly decreased tumor-induced angiogenesis (Fig 5B) and tumor growth (Fig 5C) in both MCF-7 and MDA-MB-231 xenografted CAMs.

## Discussion

The current study clearly demonstrated the signaling pathway involved in 5-HT-induced angiogenesis: 5-HT_1_ receptor-linked Src/PI3K/NOX pathway. ERK and p38 were activated in 5-HT-treated endothelial cells as previously reported [13], however, from our current study, it was clear that they are not involved in 5-HT-induced angiogenesis. In addition, we showed that 5-HT in endothelial cells can induce NOX activation and ROS production through 5-HT_1_ receptor and Gβγ, which has also been observed in other cell types including fibroblasts [42]. Furthermore, our current results showing that AZM-475271 (Src inhibitor) and wortmannin (PI3K inhibitor) suppressed 5-HT-induced ROS indicate that NOX activation by 5-HT is mediated through the 5-HT_1_ receptor-linked Src/PI3K pathway, which is consistent with observations of other types of G-protein coupled receptors (GPCRs) including CXCR2 [chemokine (C-X-C motif) receptor 2] and AT1R (angiotensin II type 1 receptors).

BJ-1108 also suppressed 5-HT-induced angiogenesis, and its antiangiogenic action was correlated with its inhibitory action on 5-HT-induced PI3K phosphorylation and superoxide generation in HUVECs. In addition to PI3K phosphorylation, PI3K enzyme activity *in vitro* was also suppressed by BJ-1108. However, BJ-1108 did not inhibit 5-HT-induced Src phosphorylation in HUVECs or Src enzyme activity *in vitro*. The results indicate that PI3K not 5-HT_1_ receptor or Src was the action site of BJ-1108. PI3K plays a critical role in angiogenesis [43, 44] as well as cancer cell proliferation, migration, invasion, survival, and malignant transformation [45], thus it is as an attractive molecular target for development of an antiangiogenic anticancer drug [46, 47]. Specifically, class I PI3K is involved in regulation of many cellular functions. Class IA PI3K consists of three isoforms of catalytic subunit, p110α, p110β, and p110δ, which are activated by receptor tyrosine kinases such as VEGFR2, whereas class IB p110γ is activated by GPCR [48]. HUVECs express p110α and p110β as major forms and p110δ at a very low level [49], and p110β is functionally redundant with p110γ in endothelial cells [48, 49]. Our current data showing that BJ-1108 suppressed PI3K p110β activity in an enzyme assay as well as 5-HT-induced PI3K phosphorylation suggest that BJ-1108 may be a broad spectrum antiangiogenic agent inhibiting a common signaling molecule downstream of receptor tyrosine kinases and GPCRs upon activation by angiogenic stimuli. Furthermore, as 5-HT-induced angiogenesis is suggested as an underlying mechanism of resistance to antiangiogenic therapy targeting VEGF due to the presence and involvement of 5-HT in the tumor microenvironment, inhibition of PI3K by BJ-1108 could be applied as an adjuvant therapy to anti-receptor tyrosine kinase therapy against cancer.

Results of the DPPH radical scavenging assay also demonstrated that BJ-1108 was a better antioxidant than vitamin C, whereas scavenging ability of BJ-1108 against superoxide produced by xanthine oxidase (XO) was lower than that by vitamin C. Although it is not clear whether BJ-1108 can inhibit enzymatic activity of XO and/or quench superoxide anion produced by XO to exert the results shown in Fig 4F, it seems clear that vitamin C is generally stronger than BJ-1108 in the inhibition of superoxide anion generation by XO. However, the two compounds showed contrasting results in DPPH radical scavenging (Fig 4E). Because of its high level of acidity (p*K*_a_ = 4.1), the majority of vitamin C exists as its anion (ascorbate) in physiological conditions at pH = 7.4. In general, anionic species exert their antioxidant mechanism mainly through electron transfer. Therefore, scavenging of DPPH radical, which is conducted via hydrogen atom transfer (HAT) from a donor molecule, by the counterpart ascorbate anion can be limited to some extent. In contrast, BJ-1108 contains the pyridinol scaffold which is known for its strong H atom transfer activity and may be the reaction with DPPH radical at lower concentration than vitamin C.

These *in vitro* antioxidant characteristics of BJ-1108 differed significantly from those of apocynin which failed to scavenge ROS in the cell-free systems (i.e., DPPH radical scavenging assay and xanthine/XO activity assay). Although BJ-1108 was a better antioxidant than apocynin, a nonspecific NOX inhibitor, in conjunction with the result that BJ-1108 did not inhibit direct activation of NOX by mevalonate or GGPP, our results indicate that the antiangiogenic action of BJ-1108 may be mediated through PI3K inhibition not through antioxidant activity.

**Fig 6:**
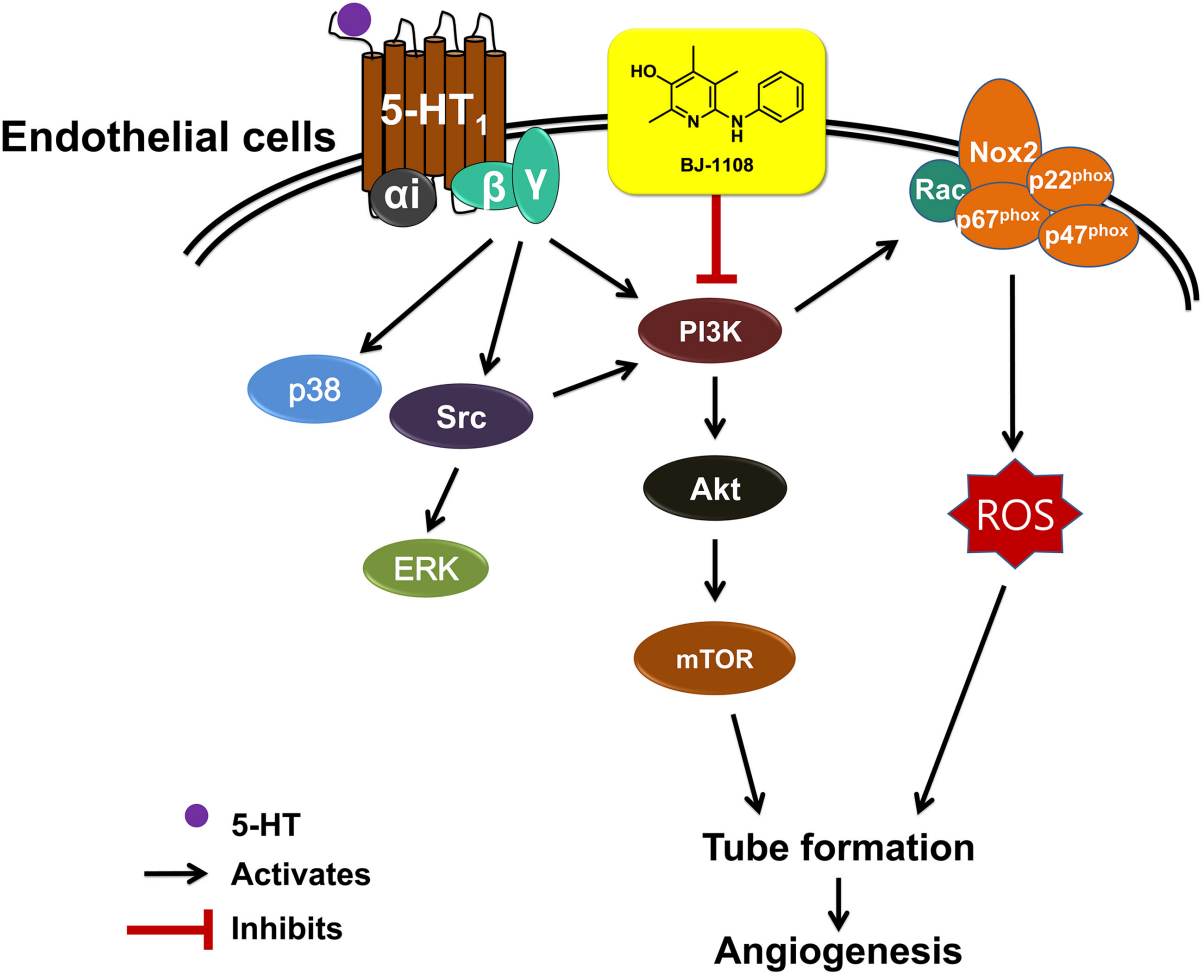
Proposed action mechanism by which BJ-1108 suppresses 5-HT-induced angiogenesis. In HUVECs, 5-HT activates Src, PI3K, NOX, AKT, mTOR, ERK and p38. However, ERK and p38 pathways are not involved in 5-HT-induced angiogenesis. BJ-1108 inhibits 5-HT-induced angiogenesis by suppressing 5-HT_1_ receptor-activated PI3K/Akt/NOX signaling pathway.

In conclusion, 5-HT induces angiogenesis through the 5-HT_1_ receptor-linked Gβγ/Src/PI3K pathway, but not through ERK or p38, and BJ-1108 targeting of the PI3K/NOX signaling pathway inhibits 5-HT-induced angiogenesis (Fig 6).
